# Time-Restricted Eating Versus Daily Calorie Restriction: Effects on Inflammatory Markers over 12 Months in Adults with Obesity

**DOI:** 10.3390/nu17071130

**Published:** 2025-03-25

**Authors:** Shuhao Lin, Sofia Cienfuegos, Mark Ezpeleta, Vasiliki Pavlou, Sarah Corapi, Mary-Claire Runchey, Shaina J. Alexandria, Lisa Tussing-Humphreys, Krista A. Varady

**Affiliations:** 1 Department of Kinesiology and Nutrition, University of Illinois Chicago, Chicago, IL 60607, USA; slin89@uic.edu (S.L.); scienf2@uic.edu (S.C.); pavlou2@uic.edu (V.P.); scorap2@uic.edu (S.C.); mrunch2@uic.edu (M.-C.R.); ltussing@uic.edu (L.T.-H.); 2 Division of Endocrinology, Metabolism and Diabetes, University of Colorado School of Medicine, Aurora, CO 80045, USA; mezpel2@uic.edu; 3 Department of Preventative Medicine (Biostatistics), Northwestern University, Chicago, IL 60208, USA; shaina.alexandria@northwestern.edu

**Keywords:** intermittent fasting, time-restricted eating, calorie restriction, weight loss, obesity, inflammation, tumor necrosis factor alpha (TNF-alpha), interleukin-6 (IL-6), C-reactive protein (CRP)

## Abstract

**Background/Objectives**: Obesity is associated with chronic systemic inflammation and elevated levels of inflammatory cytokines such as tumor necrosis factor alpha (TNF-alpha), interleukin-6 (IL-6), and C-reactive protein (CRP). Weight loss through lifestyle interventions can reduce inflammation in adults with obesity. Time-restricted eating (TRE) and calorie restriction (CR) are two popular diet interventions that can produce clinically significant weight loss. However, to date, no studies have directly compared the effects of TRE versus CR on inflammatory cytokines in adults with obesity. **Methods**: Here, we performed a secondary analysis on a recently published study to compare the long-term (12-month) effects of TRE versus CR on key inflammatory cytokines. **Results**: We found that while TRE and CR produced similar amounts of weight loss (4–5% from baseline), no statistically significant changes in circulating levels of TNF-alpha, IL-6, and CRP were noted in the TRE or CR groups, compared to the controls, by month 12. However, we did observe that circulating CRP levels were positively related to body weight, visceral fat mass, and insulin resistance, while IL-6 and TNF-alpha were not related to any metabolic marker. **Conclusions**: Thus, TRE and CR may not affect key inflammatory mediators with 4–5% weight loss, but more research is warranted.

## 1. Introduction

Obesity is associated with oxidative stress and chronic systemic low-grade inflammation [1]. An increased influx of nutrients can lead to the augmented production of reactive oxygen species (ROS), inducing oxidative stress. In addition, the accumulation of excess white adipose tissue can lead to increased macrophage recruitment and augmented hypoxia, which can in turn increase inflammatory cytokine concentrations in plasma [1]. Elevated levels of inflammatory mediators, such as tumor necrosis factor alpha (TNF-alpha), interleukin-6 (IL-6), and C-reactive protein (CRP), are observed in obesity [1]. In both animal models and humans, elevated inflammatory cytokines have been shown to interrupt the insulin signaling pathway, contributing to insulin resistance and the development of type 2 diabetes [2].

Weight loss through dietary interventions can improve chronic inflammation in adults with obesity [3]. Dietary interventions normally involve alterations of the energy content (i.e., calories restriction), or the macronutrient compositions (i.e., low-fat or high-carbohydrate diets). Among those, calorie restriction (CR) diets, where individuals are instructed to reduce their energy intake by approximately 15–40% per day, are the most commonly implemented diet strategies to achieve weight loss [4]. Previous studies have shown that moderate CR (~25% restriction) can reduce circulating inflammatory markers such as TNF-alpha, IL-6, and CRP [5,6]. However, adherence to CR diminishes over time, as many individuals find daily calorie tracking difficult to maintain for more than a few months. Thus, whether these reductions in inflammation are maintained long-term with CR remains unclear.

Time-restricted eating (TRE) has gained popularity as an alternative diet regimen to CR for weight management. TRE involves shortening an individual’s eating window to 4–10 h per day, and fasting for the rest of the day [7]. Accumulating evidence shows that TRE induces 3–5% weight loss over 2 to 12 months in adults with obesity [8]. It has also been hypothesized that by limiting the eating window to certain times of the day, TRE can synchronize central and peripheral circadian clocks. In this way, TRE may provide additional metabolic benefits such as reducing chronic inflammation [9]. To date, only a handful of trials have examined the effect of TRE on inflammatory markers in humans [10]. While some studies show that TRE reduces plasma inflammatory markers [11,12], most studies show no effect of TRE on circulating TNF-alpha, IL-6, and CRP concentrations [13]. However, these trials are limited by small sample sizes (*n* = 8–58 participants), short intervention durations (1–3 months), and the lack of a randomized controlled design. Moreover, no studies to date have directly compared the effects of TRE to a standard care diet (i.e., CR) on inflammatory markers.

We recently published a 12-month randomized controlled trial to investigate the long-term effects of TRE versus CR on body weight and metabolic risk. We reported that both TRE and CR lead to similar reductions in energy intake (~400 kcal/day) and weight loss (4–5%) versus controls. However, we did not analyze the effects of these two diet strategies on inflammation in our original study. Therefore, the aim of this secondary analysis was to compare the effects of TRE versus CR on key inflammatory markers (TNF-alpha, IL-6, and CRP) in adults with obesity over 12 months. We hypothesized that the TRE and CR interventions would have similar beneficial effects on circulating TNF-alpha, IL-6, and CRP concentrations by month 12 versus the controls.

## 2. Materials and Methods

### 2.1. Study Population

This is a secondary analysis of a previously published randomized controlled trial [14]. This study was conducted at the University of Illinois, Chicago. Participants were recruited through posters placed around the city of Chicago. Inclusion and exclusion criteria were previously reported. In brief, participants were included in the study if they were healthy adults aged between 18 and 65 with a BMI of 30 to 50 kg/m^2^. The experimental protocol was approved by the Office for the Protection of Research Participants at the University of Illinois, Chicago, USA. Written informed consent was obtained from all volunteers.

### 2.2. Experimental Design

The 12-month trial included a 6-month weight loss period followed by a 6-month weight maintenance period. During the 6-month weight loss phase, participants in the TRE group were instructed to eat ad libitum from 12 to 8 pm daily (8 h eating window) and fast from 8 to 12 pm the next day (16 h fasting window). During the 8 h eating window, there was no restriction on the amount or type of foods consumed. The CR group was instructed to limit their calories by 25% of their total daily energy needs (estimated by the Mifflin St Jeor equation [15]). At the beginning of the study, the CR participants met with the study dietitian to discuss their calorie goals and sample meal plans. Both TRE and CR participants met with the study dietitian over Zoom or phone call during the first 3 months and then biweekly from months 4 to 12. The topics covered in the counseling sessions included how to read food labeling and choose healthier food options, as well as helpful behavioral strategies for weight management.

During the 6-month weight maintenance phase, the TRE group was instructed to expand their eating window to 10 am–8 pm (10 h eating window) and fast for the rest of the day (14 h fasting window). The CR group was instructed to consume 100% of their newly calculated energy needs during the weight maintenance phase.

Throughout the whole study, the control group was asked to maintain their weight over 12 months by not changing their eating and activity habits. Dietitians contacted the controls at the same frequency as the TRE and CR groups to provide body weight measurements.

### 2.3. Outcome Measurements

#### 2.3.1. Body Weight and Body Composition

All outcomes were assessed at baseline and month 12. The primary outcome of the study was a change in body weight by month 12. Body weight was measured by a digital scale at the research center. Height was assessed during the screening visit using a wall-mounted stadiometer to the nearest 0.1 cm. Body composition (i.e., fat mass, lean mass, and visceral fat mass) was measured using dual X-ray absorptiometry (DXA; iDXA, GE; Chicago, IL, USA).

#### 2.3.2. Dietary Intake and Physical Activity

Dietary intake was assessed by a 7-day food record at baseline and month 12 using the Automated Self-Administered 24 h (ASA-24) diet assessment [16]. Physical activity (steps/day) was measured using a pedometer (Fitbit Alta, San Franciso, CA, USA) continuously for 7 days at baseline and month 12.

#### 2.3.3. Inflammatory Markers

Venous blood samples were collected via venipuncture after 12 h of fasting. Subjects were asked to avoid caffeine and exercise before the blood draw. The venous blood sample was centrifuged for 20 min at 520× *g* and 4 °C to isolate plasma. Plasma was stored at −80 °C until it was analyzed. The plasma concentrations of TNF-alpha, IL-6, and CRP were measured by an ELISA (R&D Systems, Minneapolis, MN, USA) on a Bio Rad Microplate reader (Bio-Rad Laboratories; Hercules, CA USA).

### 2.4. Quantification and Statistical Analysis

#### 2.4.1. Power and Sample Size

Sample size was calculated based on the primary outcome, change in body weight at month 12, as previously described [14]. In short, our power calculation indicated that 26 participants per group would provide us with 90% to detect a significant difference in body weight (primary outcome) between three groups with an alpha of 0.05 and effect size of 0.4125. Assuming a dropout rate of 12%, we aimed to recruit 90 participants in total.

#### 2.4.2. Randomization

A stratified randomization procedure (based on sex, age, and BMI) was used to randomize eligible participants into three groups: TRE, CR, or control.

#### 2.4.3. Statistical Analyses

The results are shown as mean values with 95% confidence intervals (CIs), unless specified otherwise. An intention-to-treat approach was used, including data from all 90 participants randomized in the study. A linear mixed model was used to evaluate time, group, and time-by-group interaction effects for each outcome. These models estimated time and group effects, along with their interaction, without imposing a linear trend over time. For pairwise comparisons of absolute changes in body weight (the primary outcome), statistical significance was determined using a Bonferroni-adjusted two-tailed *p* value threshold of <0.017. *p* values for secondary outcomes were not corrected for multiple comparisons and are presented descriptively. Correlations between absolute changes in inflammatory markers and metabolic disease risk factors were assessed using the Pearson or Spearman methods, depending on data distribution. All statistical analyses were conducted using R software (version 4.3.3).

## 3. Results

### 3.1. Participants

**Figure 1:**
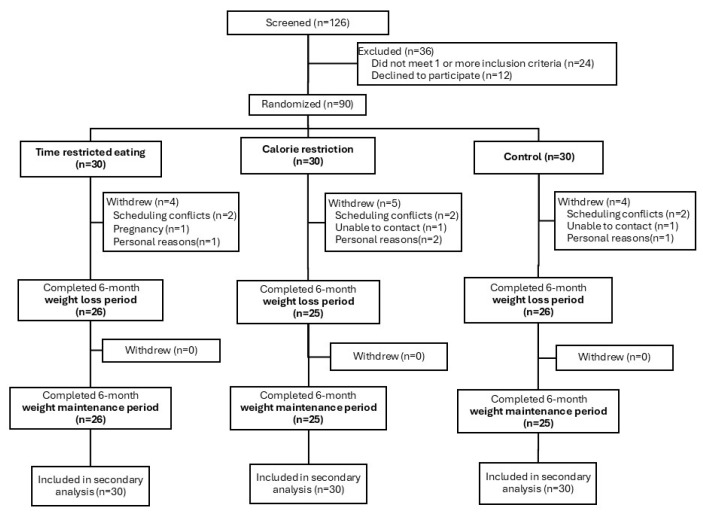
Study flow diagram.

Out of the 126 participants screened, 36 participants were excluded based on the inclusion criteria and 90 participants were randomized to the TRE, CR, or control groups (*n* = 30 in each group). Out of the 90 participants, 77 participants completed the study. The main reasons for participant attrition were scheduling conflicts, personal reasons, and unable to contact. All 90 participants who were randomized were included in the analysis (Figure 1). The baseline characteristics of the participants are shown in Table 1. The participants were primarily middle-age, Black and Hispanic women with insulin resistance. All baseline characteristics were similar between groups.

### 3.2. TRE and CR Led to Similar Changes in Body Weight Versus Controls

Changes in body weight and body composition from baseline to month 12 are shown in Table 2. As previously reported [14], participants lost −4.61 kg (CI: −7.37 to −1.85 kg) of body weight in the TRE group and −5.42 kg (CI: −9.13 to −1.71 kg) of body weight in the CR group compared to the controls. However, weight loss was not significantly different between the TRE and CR groups (0.81 kg (CI: −3.07 to 4.69 kg)) by the end of the trial. Fat mass decreased in the TRE group only (−2.77 kg (CI: −5.10 to −0.43 kg)) compared to the controls. Lean mass decreased in the CR group only (−1.13 kg (CI: −2.24 to −0.01 kg)) versus the control group. Waist circumference decreased in the TRE group only (−4.98 cm (CI: −8.09, −1.87 cm)) versus the control group. Changes in visceral fat mass did not differ between the TRE, CR, or control groups by month 12.

### 3.3. TRE and CR Produced Comparable Energy Restriction and Diet Adherence Versus Controls

Changes in dietary intake and adherence were reported in detail previously [14]. Briefly, both the TRE and CR groups reduced energy intake versus the controls (TRE: −425 ± 531 kcal/d; CR: −405 ± 712 kcal/d). TRE participants reported being adherent to their prescribed eating window on 6.1 ± 0.8 d/week throughout the 12-month study, while 61% of the CR participants reported being adherent to their prescribed calorie goals over 12 months. The dietary intakes of protein, fat, carbohydrates, total sugar, saturated fat, and fiber did not differ over time or between groups by month 12.

### 3.4. Inflammatory Markers Did Not Change in TRE or CR Groups Versus Controls over 12 Months

Changes in inflammatory markers are shown in Table 2. Changes in circulating IL-6, TNF-alpha, and CRP levels did not differ between the TRE, CR, and control groups by month 12. Sensitivity analyses were conducted for IL-6 and TNF due to the right skew of these distributions. The results from these analyses (i.e., non-parametric tests, logistic regression, log transformation) were similar to the primary results reported in Table 2.

### 3.5. Reductions in CRP Were Related to Decreases in Body Weight, Visceral Fat Mass, and Insulin Resistance

**Figure 2:**
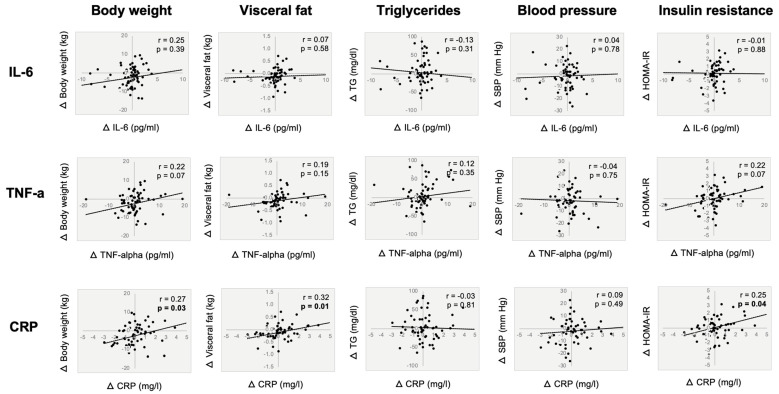
Correlations between changes in inflammatory markers and key metabolic risk factors by month 12. Correlation analyses between absolute change (baseline to month 12) in inflammatory markers and key metabolic risk factors. Data were included for 90 participants. Abbreviations: SBP: systolic blood pressure; CRP: C-reactive protein; IL-6: interleukin-6; HOMA-IR: homeostasis model assessment of insulin resistance; TNF-alpha: tumor necrosis factor alpha; TG: triglycerides.

Figure 2 depicts the correlation analyses between absolute change (baseline to month 12) in inflammatory markers and key metabolic risk factors in all subjects. Changes in CRP were positively correlated to changes in body weight (r = 0.27, *p* = 0.03), visceral fat mass (r = 0.32, *p* = 0.01), and insulin resistance (r = 0.25, *p* = 0.04). Changes in IL-6 and TNF-alpha were not related to any metabolic measure.

## 4. Discussion

To our knowledge, this is the first randomized controlled trial to compare the effects of TRE to a standard care diet (i.e., CR) on inflammatory markers. We found that while TRE and CR produced similar amounts of weight loss (~4–5% from baseline), neither diet produced significant changes in circulating levels of IL-6, TNF-alpha, or CRP, when compared to the controls, over the 12-month intervention period. However, when the group was analyzed as a whole, we did observe some relationships between reduced CRP levels and lower body weight, visceral fat mass, and insulin resistance.

Chronic low-grade inflammation and elevated cytokines levels have been proposed to be one of the causes of insulin resistance in obesity [2]. TNF-alpha is one of the most well studied inflammatory cytokines. In in vitro studies and in animal models, TNF-alpha actives c-JUN N-terminal kinase (JNK) 1 and IκB kinase (IKK), which in turn inhibit insulin receptor activation, which can lead to insulin resistance [17,18]. In humans, the infusion of TNF-alpha has been found to decrease muscle insulin sensitivity and glucose uptake, providing evidence of its role in the development of type 2 diabetes [19]. The effects of CR diets on circulating TNF-alpha concentrations have been widely studied [20]. For example, a recent study by Meydani et al. [6] reported reduced TNF-alpha levels after 24 months of 25% CR compared to the controls, with 11% weight loss. Montefusco et al. [21]. reported similar reductions in plasma TNF-alpha in participants who lost >5% body weight (mean weight loss: 9%). However, reductions in TNF-alpha seem to be highly dependent on the amount of weight loss achieved. A recent systematic review found that at least 10% weight loss may be needed to see consistent reductions in TNF-alpha levels [20]. To date, the effect of TRE on TNF-alpha concentrations has only been examined in a few studies [13]. A recent systematic review and meta-analysis found no effect of TRE on TNF-alpha concentrations when compared to no-intervention controls [13]. This is not surprising since most TRE trials report only mild-to-moderate weight loss (3–5% from baseline), which may not be sufficient to observe improvements in circulating TNF-alpha levels. Only the study by Moro et al. [11] reported decreases in TNF-alpha concentrations after 12 months of TRE plus resistance training. However, this study was carried out in healthy adults, and resistance training has been shown to improve TNF-alpha concentrations, independent of diet or weight loss [22], so this should be taken into consideration when interpreting these findings. In our study, we observed no changes in TNF-alpha concentrations in both the TRE and CR groups with a moderate amount of weight loss (~4–5%). This result is consistent with the current literature suggesting that at least 8–10% weight loss may be needed to significantly lower TNF-alpha levels in adults with obesity.

Interleukin-6 (IL-6) is another key cytokine in obesity-induced low-grade inflammation. Augmented IL-6 concentrations have been correlated to a higher risk of developing type 2 diabetes [23]. In adipocytes, IL-6 can be activated by TNF-alpha [24], and this can lead to impaired glucose uptake [24,25]. The exact mechanism of how IL-6 impacts insulin sensitivity remains unknown. While IL-6 appears to be essential for insulin production, elevated concentrations of this cytokine may increase insulin degradation and decrease glucose uptake [24,25]. Similarly to TNF-alpha, reductions in circulating IL-6 concentrations are usually only observed with diet interventions, resulting in 8–10% of weight loss [6,20,22]. To this point, most TRE human trials report no change in circulating IL-6 levels, since only a minimal amount of weight loss is attained (3–5% from baseline). In our study, the IL-6 concentrations did not differ between the TRE, CR, and control groups after 12 months of intervention. Thus, these diet strategies may not impact circulating IL-6 levels with only a moderate amount of weight loss. More long-term studies will be needed to confirm these findings.

CRP is an acute phase inflammatory protein secreted by the liver [26]. While it is generally activated during acute infection and inflammatory responses, it is also elevated in obesity [27]. CRP production in the liver is activated by IL-6. The elevated levels of circulating CRP in obesity are linked to an increased incidence of type 2 diabetes and cardiovascular diseases [27,28]. The effect of a caloric restriction on CRP levels depends on the amount of weight loss achieved [29]. Evidence suggests that at least 5% weight loss may be needed to see significant reductions in CRP levels, and more than 10% weight loss is required in order to see improvements consistently [29,30]. For example, Meydani et al. [6] observed significant reductions in circulating CRP levels with 11% weight loss, while Montefusco et al. [21] reported no change in CRP levels with only 9% weight loss. Studies examining the effects of TRE on plasma CRP levels report mixed findings. A couple of trials in adults with obesity have found no change in circulating CRP due to the minimal amount of weight loss attained (3–5% from baseline) [10,13]. In contrast, four other TRE trials showed reductions in CRP levels, even though the degree of weight loss did not exceed the 5% threshold [12,31,32,33]. However, it should be noted that these studies were performed in different populations (i.e., healthy young adults [33], shift workers [32], or women with polycystic ovary syndrome [31]) and/or involved alterations in dietary components (i.e., low-sugar diet [12]), which may explain why the results differed from our study. In the present trial, we did not observe any change in circulating CRP levels in either TRE or CR, versus the controls, with moderate weight loss (4–5%). This is consistent with previous reports showing that over 10% of weight loss may be needed to see improvements in the levels of this inflammatory cytokine. Nevertheless, when the sample was analyzed as a whole, we did observe that CRP was positively related to body weight, visceral fat mass, and insulin resistance. This would suggest that participants with the greatest reductions in body weight and visceral fat mass may be more likely to have lower CRP levels, which could help decrease insulin resistance. This is only a speculation, however, as these findings are only correlational, and not causational.

Our study has several limitations. First, this is a secondary analysis. Since our power analysis was based on the primary outcome of a change in body weight, it is likely that we are not adequately powered to detect changes in inflammatory markers. Second, we did not limit or monitor the intake of anti-inflammatory supplements such as omega-3 fatty acids and vitamin D, which may have confounded our results [34,35]. Third, the majority (~80%) of our participants were female, which limits the generalizability of our findings. Fourth, we only measured TNF-alpha, IL-6, and CRP levels. It is possible that other inflammatory markers (such as IL-8, ILβ), certain oxidative stress markers (such as 8-isoprostane), and adipokines (such as leptin, ghrelin, or adiponectin) may provide a greater understanding of the underlying mechanisms. Moreover, we only measured systematic inflammation by plasma cytokine concentrations in our study. However, obesity is associated with both systematic and local tissue inflammation. Therefore, it is possible that we failed to capture changes in local tissue inflammation. Lastly, we only reported changes in inflammation markers at month 12. It is possible that we missed changes in inflammatory mediators that may have occurred earlier in the study (e.g., month 3 or 6) or post-intervention.

## 5. Conclusions

In conclusion, neither TRE nor CR improved circulating inflammatory cytokine levels (i.e., TNF-alpha, IL-6, and CRP) over 12 months, compared to the controls, with 4–5% of weight loss. However, when the sample was analyzed as a whole, we did observe some relationships between reduced CRP levels and lower body weight, visceral fat mass, and insulin resistance. More well-powered studies designed to specifically examine the effects of these diets on inflammatory mediators are needed to confirm these findings.

## Figures and Tables

**Table 1:** Baseline characteristics of the study participants.

	Time-Restricted Eating	Calorie Restriction	Control
*n*	30	30	30
Age (y)	44 ± 12	44 ± 9	44 ± 13
Sex, no. (%)			
Female	25 (83%)	24 (80%)	25 (83%)
Male	5 (17%)	6 (20%)	5 (17%)
Race or ethnic group, no. (%)			
Black	11 (37%)	9 (30%)	10 (33%)
Asian	3 (10%)	3 (10%)	0 (0%)
Hispanic	13 (43%)	11 (37%)	17 (57%)
White	3 (10%)	7 (23%)	3 (10%)
Body weight and composition			
Body weight (kg)	100 ± 17	102 ± 18	102 ± 17
Fat mass (kg)	46 ± 11	47 ± 11	47 ± 10
Lean mass (kg)	50 ± 10	50 ± 9	51 ± 8
Visceral fat mass (kg)	1.6 ± 0.6	1.6 ± 0.8	1.7 ± 0.8
Waist circumference (cm)	109 ± 13	110 ± 14	110 ± 13
Height (cm)	164 ± 9	166 ± 9	165 ± 7
BMI (kg/m^2^)	37 ± 6	37 ± 5	38 ± 5
Inflammatory markers			
IL-6 (pg/mL)	4.1 ± 3.0	4.3 ± 5.2	3.2 ± 2.8
TNF-alpha (pg/mL)	9.7 ± 5.0	8.7 ± 3.8	13.7 ± 11.6
CRP (mg/L)	3.5 ± 1.4	3.3 ± 1.6	3.6 ± 1.8
Blood pressure, heart rate			
Systolic BP (mm Hg)	124 ± 16	125 ± 14	126 ± 14
Diastolic BP (mm Hg)	84 ± 10	83 ± 9	85 ± 10
Heart rate (bpm)	75 ± 12	75 ± 13	74 ± 13
Plasma lipids			
Total cholesterol (mg/dL)	185 ± 31	182 ± 37	178 ± 32
LDL cholesterol (mg/dL)	107 ± 27	110 ± 33	102 ± 28
HDL cholesterol (mg/dL)	55 ± 14	55 ± 11	49 ± 13
Triglycerides (mg/dL)	115 ± 47	88 ± 32	141 ± 75
Glucoregulatory factors			
Fasting glucose (mg/dL)	89 ± 12	88 ± 13	87 ± 12
Fasting insulin (μIU/mL)	17 ± 11	11 ± 6	17 ± 10
Insulin resistance (HOMA-IR)	3.6 ± 2.8	2.6 ± 1.4	3.6 ± 2.6
Insulin sensitivity (QUICKI)	0.33 ± 0.03	0.34 ± 0.03	0.33 ± 0.03
HbA1c (%)	5.5 ± 0.5	5.4 ± 0.5	5.5 ± 0.4

Data are expressed as mean (SD) unless otherwise indicated. Abbreviations: BP: blood pressure; BMI: body mass index; CRP: C-reactive protein; HbA1c: glycated hemoglobin; HDL: high-density lipoprotein; IL-6: interleukin-6; HOMA-IR: homeostasis model assessment of insulin resistance; LDL: low-density lipoprotein; QUICKI: quantitative insulin sensitivity check index; TNF-alpha: tumor necrosis factor alpha. Demographic data previously published [14].

**Table 2:** Changes in body weight, metabolic risk factors, and inflammatory markers by month 12.

Variables	Change from Baseline to Month 12 (95% CI)			Difference Between Groups by Month 12 (95% CI)		
	Time-Restricted Eating (TRE)	Daily Calorie Restriction (CR)	Control (CON)	TRE vs. CR	TRE vs. CON	CR vs. CON
**Body weight and composition**						
Body weight (kg)	−3.49 (−5.65, −1.32)	−4.30 (−7.63, −0.96)	1.12 (−0.69, 2.94)	0.81 (−3.07, 4.69)	−4.61 (−7.37, −1.85)	−5.42 (−9.13, −1.71)
Body weight (%)	−3.76 (−5.89, −1.64)	−4.20 (−7.59, −0.80)	1.11 (−0.72, 2.94)	0.43 (−3.48, 4.34)	−4.87 (−7.61, −2.13)	−5.30 (−9.06, −1.54)
Fat mass (kg)	−2.20 (−3.88, −0.52)	−2.61 (−5.97, 0.74)	0.57 (−1.14, 2.27)	0.42 (−3.24, 4.07)	−2.77 (−5.10, −0.43)	−3.18 (−6.85, 0.49)
Lean mass (kg)	−0.41 (−0.91, 0.08)	−0.74 (−1.44, −0.03)	0.39 (−0.51, 1.29)	0.32 (−0.52, 1.16)	−0.81 (−1.81, 0.20)	−1.13 (−2.24, −0.01)
Visceral fat mass (kg)	−0.14 (−0.23, −0.04)	−0.12 (−0.29, 0.06)	−0.03 (−0.16, 0.10)	−0.02 (−0.22, 0.17)	−0.11 (−0.27, 0.06)	−0.08 (−0.30, 0.13)
Waist circumference (cm)	−6.44 (−8.65, −4.24)	−3.77 (−7.46, −0.08)	−1.46 (−3.77, 0.84)	−2.67 (−6.86, 1.52)	−4.98 (−8.09, −1.87)	−2.30 (−6.55, 1.94)
**Inflammatory markers**						
IL-6 (pg/mL)	−1.18 (−2.29, −0.06)	−0.63 (−2.10, 0.84)	0.90 (−1.38, 3.18)	−0.55 (−2.34, 1.25)	−2.07 (−4.55, 0.40)	−1.53 (−4.17, 1.12)
TNF-alpha (pg/mL)	−0.63 (−1.95, 0.70)	1.08 (−1.20, 3.36)	−0.15 (−2.93, 2.63)	−1.71 (−4.27, 0.85)	−0.48 (−3.47, 2.52)	1.23 (−2.27, 4.72)
CRP (mg/L)	0.11 (−0.63, 0.84)	−0.13 (−0.74, 0.47)	0.22 (−0.31, 0.75)	0.24 (−0.69, 1.17)	−0.11 (−1.00, 0.77)	−0.35 (−1.14, 0.43)
**Blood pressure, heart rate**						
Systolic BP (mm Hg)	−1.78 (−6.80, 3.24)	−4.62 (−8.92, −0.31)	0.06 (−4.52, 4.64)	2.84 (−3.62, 9.29)	−1.84 (−8.47, 4.79)	−4.68 (−10.81, 1.46)
Diastolic BP (mm Hg)	−0.82 (−4.70, 3.05)	0.99 (−2.13, 4.10)	2.85 (0.10, 5.59)	−1.81 (−6.66, 3.04)	−3.67 (−8.30, 0.97)	−1.86 (−5.91, 2.18)
Heart rate (bpm)	−3.83 (−8.31, 0.64)	1.63 (−3.32, 6.59)	−2.74 (−7.06, 1.59)	−5.47 (−11.98, 1.05)	−1.10 (−7.17, 4.98)	4.37 (−2.05, 10.79)
**Plasma lipids**						
Total cholesterol (mg/dL)	−1.69 (−9.73, 6.36)	−1.64 (−9.80, 6.52)	−0.67 (−7.37, 6.03)	−0.05 (−11.21, 11.11)	−1.02 (−11.22, 9.18)	−0.97 (−11.25, 9.31)
LDL cholesterol (mg/dL)	−0.86 (−9.14, 7.42)	−1.33 (−7.47, 4.81)	2.37 (−4.51, 9.24)	0.47 (−9.57, 10.52)	−3.22 (−13.71, 7.26)	−3.70 (−12.67, 5.28)
HDL cholesterol (mg/dL)	−1.78 (−5.04, 1.48)	1.01 (−3.17, 5.19)	−0.76 (−2.76, 1.23)	−2.79 (−7.96, 2.38)	−1.02 (−4.74, 2.71)	1.77 (−2.74, 6.29)
Triglycerides (mg/dL)	3.60 (−10.22, 17.42)	−0.02 (−12.85, 12.81)	−0.08 (−17.95, 17.78)	3.62 (−14.76, 21.99)	3.68 (−18.33, 25.69)	0.07 (−21.36, 21.49)
**Glucoregulatory factors**						
Fasting glucose (mg/dL)	2.82 (−1.15, 6.79)	5.83 (1.25, 10.40)	6.26 (1.63, 10.90)	−3.01 (−8.91, 2.90)	−3.44 (−9.39, 2.51)	−0.43 (−6.78, 5.91)
Fasting insulin (μIU/mL)	−2.83 (−5.34, −0.33)	−0.12 (−2.22, 1.97)	1.44 (−2.32, 5.21)	−2.71 (−5.89, 0.48)	−4.27 (−8.68, 0.13)	−1.57 (−5.77, 2.63)
Insulin resistance (HOMA-IR)	−0.49 (−1.23, 0.24)	0.07 (−0.47, 0.61)	0.54 (−0.33, 1.41)	−0.56 (−1.45, 0.32)	−1.03 (−2.14, 0.07)	−0.47 (−1.46, 0.53)
Insulin sensitivity (QUICKI)	0.01 (0.00, 0.02)	0.00 (−0.02, 0.01)	−0.01 (−0.02, 0.00)	0.01 (0.00, 0.03)	0.02 (0.01, 0.04)	0.01 (−0.01, 0.02)
HbA1c (%)	0.00 (−0.15, 0.14)	0.05 (−0.07, 0.18)	0.07 (−0.03, 0.18)	−0.06 (−0.25, 0.13)	−0.08 (−0.25, 0.10)	−0.02 (−0.18, 0.14)

Data were included for 90 participants; means were estimated using an intention-to-treat analysis using a linear mixed model. Error bars indicate 95% confidence intervals for each parameter from baseline by diet group. Abbreviations: BP: blood pressure, CON: control, CR: calorie restriction, CRP: C-reactive protein, HbA1c: glycated hemoglobin, HDL: high-density lipoprotein, IL-6: interleukin-6, HOMA-IR: homeostasis model assessment of insulin resistance, LDL: low-density lipoprotein, QUICKI: quantitative insulin sensitivity check index, TNF-alpha: tumor necrosis factor alpha, TRE: time-restricted eating. Body weight and body composition data were previously reported in [14].

## Data Availability

The original contributions presented in this study are included in the article; further inquiries can be directed to the corresponding authors.
